# Characterization of a shorter recombinant polypeptide chain of bone morphogenetic protein 2 on osteoblast behaviour

**DOI:** 10.1186/s12903-015-0154-z

**Published:** 2015-12-30

**Authors:** Yufeng Zhang, Yang Shuang, Hang Fu, Wei Zhou, Li Qian, Jing Dai, Richard J. Miron

**Affiliations:** The State Key Laboratory Breeding Base of Basic Science of Stomatology (Hubei-MOST) & Key Laboratory of Oral Biomedicine Ministry of Education, School & Hospital of Stomatology, Wuhan University, 237 Luoyu Road, Wuhan, 430079 People’s Republic of China; Department of Oral Implantology, School of Stomatology, Wuhan University, Wuhan, 430079 China; Department of Oral Surgery and Stomatology, University of Bern, Freiburgstrasse 7, Bern, 3010 Switzerland; Department of Periodontology, School of Dental Medicine, University of Bern, Freiburgstrasse 7, Bern, 3010 Switzerland; Hangzhou JIuyuan Gene Engineering Co, Ltd;East of No.8 Street, Hangzhou Econ. and Tech. Development Zone, Hangzhou, China Hangzhou China 310018 China

**Keywords:** BMP2, Osteoinductive, Osteoinduction, Osteoblast differentiation, Guided bone regeneration

## Abstract

**Background:**

Recombinant bone morphogenetic protein two (rhBMP2) has been utilised for a variety of clinical applications in orthopaedic surgery and dental procedures. Despite its widespread use, concerns have been raised regarding its short half-life and transient bioactivity in vivo. Recent investigation aimed at developing rhBMP2 synthesized from a shorter polypeptide chain (108 amino acids) has been undertaken.

**Methods:**

The osteopromotive properties of BMP2 were investigated on cell behaviour. Five concentrations of rhBMP2_108 including 10, 50, 100, 200 and 500 ng/ml were compared to a commercially available rhBMP2 (100 ng/ml). Each of the working concentrations of rhBMP2_108 were investigated on MC3T3-E1 osteoblasts for their ability to induce osteoblast recruitment, proliferation and differentiation as assessed by alkaline phosphatase (ALP) staining, alizarin red staining, and real-time PCR for genes encoding ALP, osteocalcin (OCN), collagen-1 (COL-1) and Runx2.

**Results:**

The results demonstrate that all concentrations of rhBMP2_108 significantly improved cell recruitment and proliferation of osteoblasts at 5 days post seeding. Furthermore, rhBMP2_108 had the most pronounced effects on osteoblast differentiation. It was found that rhBMP2_108 had over a four fold significant increase in ALP activity at seven and 14 days post-seeding and the concentrations ranging from 50 to 200 ng/ml demonstrated the most pronounced effects. Analysis of real-time PCR for genes encoding ALP, OCN, COL-1 and Runx2 further confirmed dose-dependant increases at 14 days post-seeding. Furthermore, alizarin red staining demonstrated a concentration dependant increase in staining at 14 days.

**Conclusion:**

The results from the present study demonstrate that this shorter polypeptide chain of rhBMP2_108 is equally as bioactive as commercially available rhBMP2 for the recruitment of progenitor cells by facilitating their differentiation towards the osteoblast lineage. Future in vivo study are necessary to investigate its bioactivity.

## Background

The use of growth factors and biomaterials with bone inducing properties has played a pivotal role in treatment options for patients suffering from a variety of bone defects caused by either fracture or disease [1–3]. Osteoporosis, a disease characterized by low mineral density and subsequent fragility [4] has now reached an estimated 200 million people worldwide with 80 % being post-menopausal women [5–7]. The significant increase in bone metabolic diseases in combination with traumatic injuries caused by sports accidents, motor vehicle accidents and other related fractures necessitates bone inducing agents capable of speeding up bone regeneration often in compromised scenarios such as osteoporotic related fractures [8–11].

One growth factor that has been widely used with FDA approval is that of bone morphogenetic proteins (BMPs) [12–14]. BMPs were initially extracted from demineralized bone matrix and it was revealed that these low-molecular weight proteins demonstrated the ability to form ectopic bone in extra-skeletal sites in animals [15, 16]. Since then, BMPs have been capable of regenerating a multitude of osseous defects and have been shown to improve cell recruitment, proliferation and differentiation of mesenchymal cells, and speeding up their differentiation towards bone forming osteoblasts [17]. Despite the clinical advantages of recombinant proteins, concerns have been raised regarding their transient bioactivity, protein instability, fast dissolution rates and short half lives [18]. For these reasons, the use of rhBMPs are typically administered in high supra-physiological doses that bear the risk of potential side effects associated with their use [19]. Furthermore, other factors that could lead to a reduced in vivo bioactivity include the role of antagonists and their specificity for different BMPs (noggin, chordin, dan), the role of the host organism for expression (E.coli vs. CHO) and the associated differences in glycosylation patterns that might lead to differences in bioavailability and stability [20–22].

Recently the development of a novel recombinant protein fabricated from a shorter polypeptide chain of BMP2 has been undertaken with the aim of improving protein stability in vivo. This shorter protein chain is hypothesized to facilitate protein folding, thus potentially improving the bioactivity of rhBMP2 by increasing the half-life of the protein [23]. However, prior to animal testing, a full characterization of this shorter amino acid sequence was investigated on cell behaviour in vitro. MC3T3-E1 pre-osteoblasts were investigated for their response to five different concentrations of rhBMP2_108 including 10, 50, 100, 200 and 500 ng/ml and compared to control commercially available rhBMP2 at a concentration of 100 ng/ml (R&D systems). rhBMP2_108 was tested for its ability to induce cell recruitment, cell proliferation, alkaline phosphatase activity, alizarin red staining as well as mRNA levels for genes encoding alkaline phosphatase, osteocalcin, collagen one and Runx2 as assessed by real-time PCR.

## Methods

### rhBMP2_108 amino acid sequence

rhBMP2_108 was kindly provided by Jiuyuan biotech company (Hangzhou, China). The shortened amino acid sequence was fabricated as follows: MKKLKSSCKRHPLYVDFSDVGWNDWIVAPPGYHAFYCHGECPFPLADHLNSTNHAIVQTLVNSVNSKIPKACCVPTELSAISMLYLDENEKVVLKNYQDMVVEGCGCR for a protein chain of 108 amino acids. The commercially available BMP2 was purchased from R&D systems (Minneapolis, USA).

### Preparation of BMP2

According to the amino acid sequence, intracellular expression of rhBMP2_108 in E. coli was constructed for bulk production of rhBMP2_108 as previously described [24]. Briefly, the sequence of BMP2 was designed according to its amino acid sequence, and then was reverse transcribed with reverse transcriptase (Gibco BRL, NY, USA) as previously described [24]. Polymerase chain reaction (PCR) was used to amplify the cDNA encoding the shortened amino acid sequence rhBMP2_108 protein. This rhBMP2_108 cDNA was then subcloned using a pRSET(A) vector (Invitrogen, UK) to yield the pRSET(A)/rhBMP2_108 expression vector. Then, pRSET(A)/rhBMP2_108 was further utilized to transform the E. coli BL21(DE3) strain. A bioreactor was then utilized to yield high cell-density cultivation of E. coli. Briefly, transformed E. coli was cultured in a 5 L fermenter, with 3 L of the defined medium (batch culture; yeast extract 1 g/L, peptone 2 g/L) inoculated with the pre-culture (10 % of the batch culture volume). The cultivation was performed with stirring at 250 rpm for 48 h at 30 °C, including the addition of a nutrient medium (glucose 33.3 g/L, peptone 10 g/L, yeast extract 5 g/L,MgSO_4_1 g/L, FeSO_4_8.0 g/L, CaCl_2_0.048 g/L, ZnSO_4_0.0176 g/L, CuSO_4_0.008 g/L).

The cultured biomass was then harvested by crushing twice the cells in a French press and thereafter centrifuged. The pellet was resuspended at 25 mg wet weight/ml in a suspension buffer (20 mMTris–HCl [pH 8.5], 0.5 mMEDTA, 2 % [v/v]Triton X-100). The inclusion bodies (pellets) were resuspended in a solubilization buffer (6Mguanidine-HCl, 0.1 M Tris–HCl [pH 8.5],0.1 M DTT, 1 mM EDTA) and thereafter incubated with constant stirring at room temperature overnight. Insoluble particles were then removed from the suspension by centrifugation.

A Heparin Sepharose 6 Fast Flow column was then used to purify the active rhBMP2_108 (dimer). A continuous NaCl gradient (0.1–1.5 M) was used to elute the bound protein. Afterwards, a stepped NaCl gradient (0.15 M, 0.3 M, and 0.5 M) was used to elute and separate the active rhBMP2_108 protein. Thereafter rhBMP2_108 powder was frozen at−20 °C. When used, 0.1 % acetic acid was utilized to dissolve the BMP2 powder. Control rhBMP2 was purchased from R & D systems (Minneapolis, USA) derived from CHO cells.

### Two-dimensional migration assay

MC3T3-E1 pre-osteoblast cell line was used for this study. No human or animal samples were utilized and thus no ethical consent was required. The migration assay of cells was performed with a Transwell chamber using a 24-well plate and polycarbonate filters (Transwell Costar, Corning, Acton, MA) with a pore size of 8 μm as previously described [25]. 10^4^ MC3T3-E1 cells in 50 μl DMEM were seeded in the upper compartment. rhBMP2_108 at concentrations of 0, 10, 50, 100, 200 and 500 ng/ml and control rhBMP2 at 100 ng/ml were seeded into the lower compartment. The cells were allowed to migrate for 24 h at 37 °C in a humidified 5 % CO_2_ atmosphere. The filter was then removed, cells were fixed in 4 % formaldehyde for 20 min and washed in PBS, after washings, filters were incubated for 15 min at room temperature with Methylrosanilnium Chloride Solution (Wuhan Google biotechnology limited company G1014), then the filters were washed with PBS for 10 min. Samples were examined by microscopy. Non-migrated cells on the upper side were eliminated by rinsing the filter with cold PBS and scraping with a rubber wiper. The remaining migrated cells on the lower side of the filter were counted in nine random fields per filter (×100 magnification). All samples were performed in triplicate with 3 independent experiments performed.

### Proliferation assay

To investigate the effect of rhBMP2_108 on the proliferation of MC3T3-E1, CCK-8 assay for each group was performed as previously described [26]. Briefly, cells were seeded in 96-well plates at a density of 5×10^3^ cells/well. At time points 1, 3 and 5 days, the CCK-8 assay was performed by adding 10 μL of the CCK-8 solution (Dojindo Molecular Technologies, Inc. Japan) to each well and incubating for 1.5 h according to manufacturer’s protocol. The absorbance was measured at λ = 450 nm on a plate reader. Results were demonstrated as the absorbance of each experimental well minus the optical density value of blank wells. All samples were repeated in triplicate with three independent experiments.

### Alkaline phosphatase activity

Alkaline phosphatase activity was analyzed colorimetrically using alkaline phosphatase assay kit (Nanjing Jiancheng Bioengineering Insitute) using a starting seeding density of 50’000 cells per 24 well dish as previously described [27, 28]. At time point of seven and 14 days, cells were washed three times with PBS and solubilized in 0.1 % Triton X-100 (buffered in 0.1 mol/L PBS, pH 7.3) at 4 °C for 1 h. After sonication and centrifugation, ALP activity in the supernatant was determined colorimetrically using readings OD405/OD562. Total protein was quantified by BCA Protein Assay Kit (Thermo Fisher Scientific Inc.). The ALP activity was normalized to the total protein. Samples were run in triplicate with three independent experiments performed.

### Real-time PCR

MC3T3-E1 cells were seeded at a density of 2×10^5^ and cultured for 14 days as previously described [26, 28, 29]. The effects of rhBMP2_108 at various concentrations were investigated on four osteogenic-related gene expression including alkaline phosphatase (ALP), collagen I (COL I), runt-related transcription factor two (Runx 2) and osteocalcin (OCN). Total RNA was extracted from MC3T3-E1 cells by Trizol reagent (TriPure Isolation Reagent, Roche Applied Science, Germany) according to the manufacturer’s instructions. The concentration and quality of the total RNA samples was analyzed by Nanodrop (Thermo Fisher Scientific Inc.). Complementary DNA was synthesized from 2 μg of total RNA using RevertAidTm First Strand cDNA Synthesis Kit (Fermentas) following the manufacturer’s protocol. RT-qPCR was performed in 20 μL reactions containing 10 μl SYBR Green Master Mix (Roche Applied Science, Germany), 0.6 μL (10 μM) of each forward and reverse primer for each gene of interest, 2 μL of cDNA template and 6.8 μL water. The primer sequences (shown in Table 1) have designed specifically according to the reference. Glyceraldehyde-3-phosphate-dehydrogenase (GAPDH), a reference gene, was used as a control. The reaction was analyzed using an ABI Prism 7000 Sequence Detection System (Applied Biosystems), and the PCR amplification run for 40 cycles. To validate specific amplicon amplification without genomic DNA contamination, melting curve analysis was performed and the single apex appeared around the annealing temperature. Relative expression levels for each desired gene were normalized against the Ct value of GAPDH and determined by using the delta Ct method. All samples were determined in triplicate for three independent studies.

**Table 1:** Primer sequences for osteoblast differentiation markers

GAPDH F	GTGAAGGTCGGTGTGAACGG
GAPDH R	TCCTGGAAGATGGTGATGGG
OPN F	GAGGAAACCAGCCAAGGTAAG
OPN R	AAAGCAAATCACTGCCAATCTC
OCN F	GAGGACCATCTTTCTGCTCACT
OCN R	CGGAGTCTGTTCACTACCTTATTG
ALP F	TGTGGAATACGAACTGGATGAG
ALP R	ATAGTGGGAATGCTTGTGTCTG
RUNX2 F	GTGTTCCCTACTCAGCCGTC
RUNX2 R	GAGGCCTCGGTCCACATTAG

### Alizarin red quantification

Alizarin red staining was performed to determine the presence of extracellular matrix mineralization after 14 days. MC3T3-E1 cells were seeded at a density of 50,000 cells per 24 well culture dish containing the various concentrations of rhBMP2_108. After 14 days, cells were fixed in 96 % ethanol for 15 min and stained with 2 % alizarin red solution in water (pH 4.2) at room temperature for 1 h and visualized under light microscopy. Thereafter, alizarin red quantification was dissolved using 200 μl of 1 % cetylpyridinium chloride (dissolved with double distilled water) at room temperature for 4 h. Then 100 μl solution was transferred to 96-well plate to text OD 560.

### Statistical analysis

All data analysis was performed using GraphPad Prism6.0 software. Normal distribution of sample data was assumed for the current study and therefore parametric testing was analyzed using one-way ANOVA and statistically significant values were adopted as *p* < 0.05. Mean and standard error (SE) were analyzed using one-way ANOVA with a post hoc test with Tukey’s analysis.

## Results

### Effect of rhBMP2_108 on cell recruitment

**Fig. 1:**
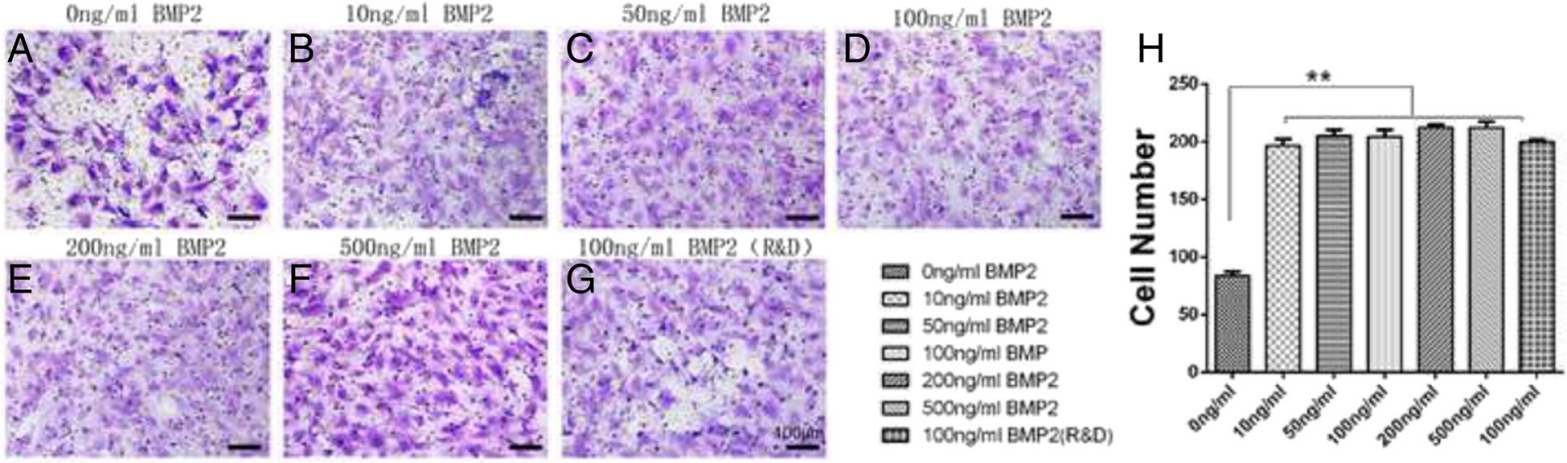
Cell recruitment assay demonstrated that all concentrations of rhBMP2_108 were significantly able to improve MC3T3-E1 cell recruitment when compared to control samples with no rhBMP2 (Scale bar = 100 μm). All experiments were conducted in triplicate with three independent experiments (*n* = 9). Data represents means +/− SEM. ** denotes all samples significantly higher than control samples, *p* < 0.01)

In order to investigate the effects of rhBMP2_108 on cell recruitment, a transwell chamber was utilised at five different concentrations of rhBMP2_108 (Fig. 1). It was found that after a 24 h period, all concentrations of rhBMP2_108 and control rhBMP2 were able to significantly increase cell recruitment of MC3T3-E1 cells when compared to control samples. Little variability between samples was observed demonstrating a strong potential for all concentrations of rhBMP2_108 to induce cell recruitment (Fig. 1).

### Effect of rhBMP2 on cell proliferation

**Fig. 2:**
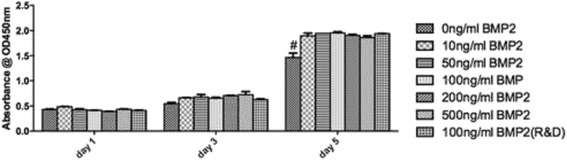
Cell number as calculated by an CCK-8 assay for MC3T3-E1 cells seeded at various concentrations of rhBMP2_108 when compared to control tissue culture plastic (All experiments were conducted in triplicate with three independent experiments (*n* = 9). Data represents means +/− SEM. # denotes control samples significantly lower than all other treatment modalities, *p* < 0.05)

The effect of rhBMP2_108 was investigated at five concentrations and control rhBMP2 for their ability to stimulate cell proliferation in vitro (Fig. 2). It was found that MC3T3-E1 cells demonstrated very similar cell number 1 day post-seeding irrespective of cell culture media concentration of rhBMP2_108 (Fig. 2). A slight increase in cell number was observed at 3 days, however, no significant difference could be observed between the various treatment modalities (Fig. 2). By 5 days post seeding, a significant increase in cell number was observed for MC3T3-E1 cells with all concentrations of rhBMP2_108 used in this study (Fig. 2). Interestingly, no advantages could be found for higher concentrations of rhBMP2_108 when compared to their lower counterparts (Fig. 2).

### Effect of rhBMP2 on cell differentiation

**Fig. 3:**
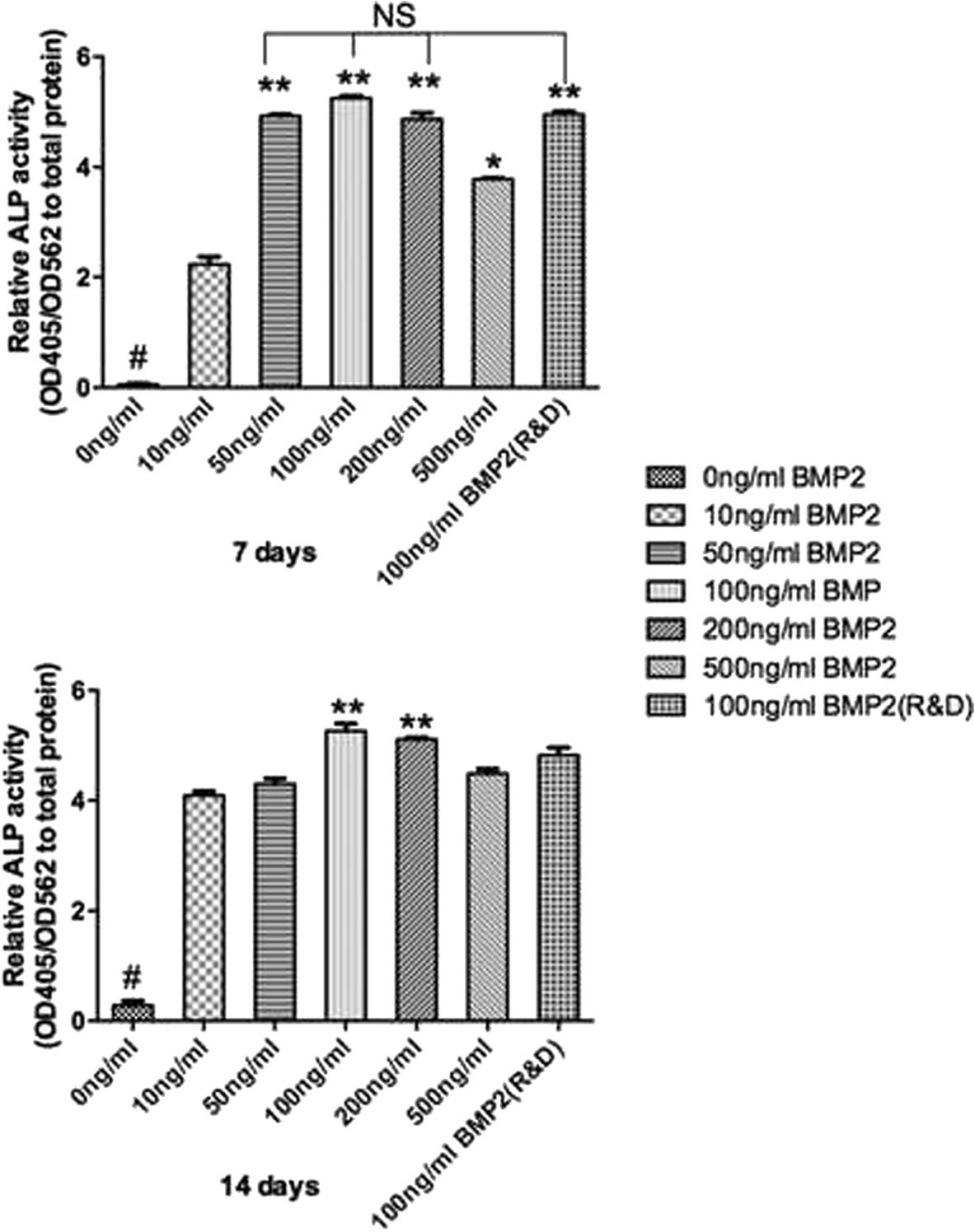
ALP activity was significantly increased at seven and 14 days post seeding for samples seeded with rhBMP2_108 when compared to control tissue culture plastic alone (All experiments were conducted in triplicate with three independent experiments (*n* = 9). Data represents means +/− SEM. * denotes significant difference between 500 ng/ml and 10 ng/ml rhBMP2, *p* < 0.05; ** denotes significantly higher than all other modalities, *p* < 0.05, # denotes control samples significantly lower than all other treatment modalities, *p* < 0.05)

**Fig. 4:**
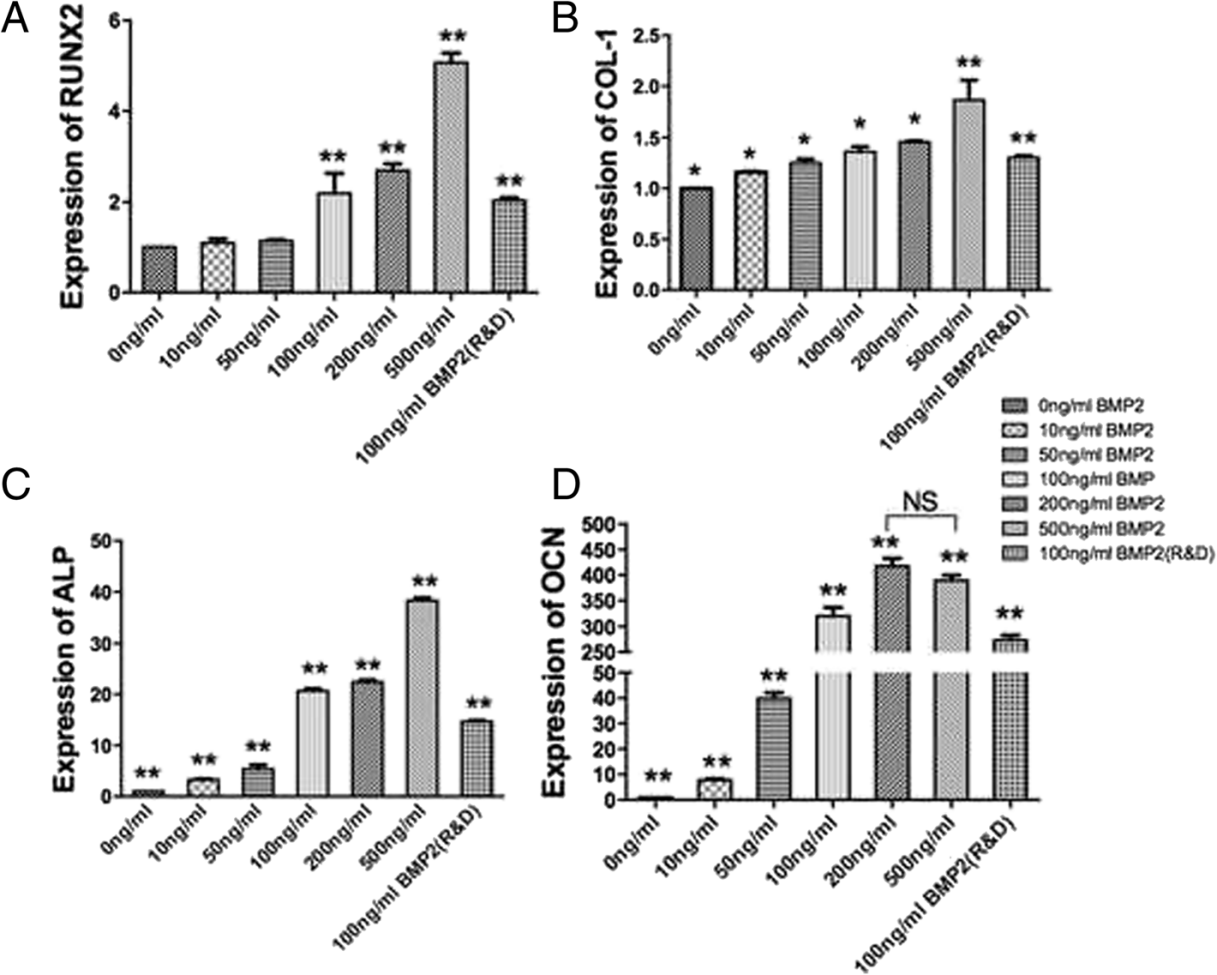
mRNA levels of (**a**) ALP, (**b**) OCN, (**c**) COL-1 and (**d**) Runx2 for MC3T3-E1 cells seeded at various concentrations of rhBMP2_108 (All experiments were conducted in triplicate with three independent experiments (*n* = 9). Data represents means +/− SEM. * denotes *p* < 0.05; ** denotes significantly higher than all other modalities, *p* < 0.05, # denotes control samples significantly lower than all other treatment modalities, *p* < 0.05)

**Fig. 5:**
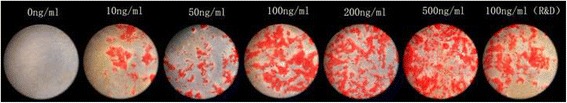
Qualitative analysis of alizarin red staining demonstrated increased mineralization for MC3T3-E1 cells at increasing concentrations of rhBMP2_108 at 14 days post seeding

**Fig. 6:**
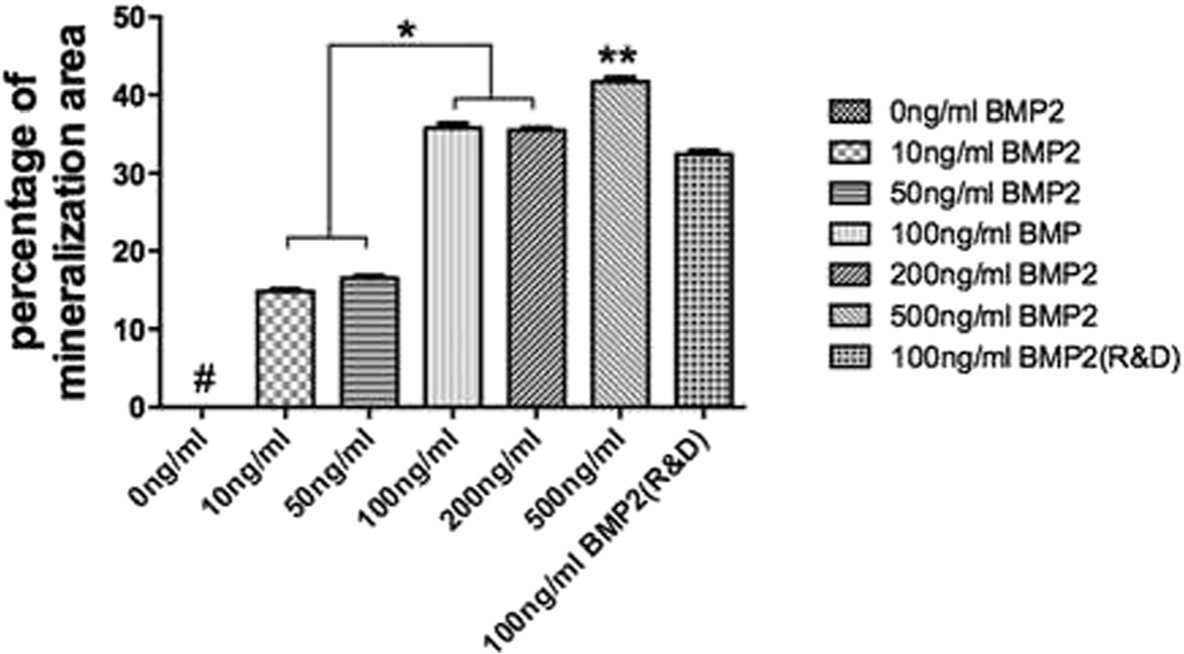
Quantitative analysis of alizarin red staining. rhBMP2_108 demonstrate a concentration dependent increase in alizarin red staining (All experiments were conducted in triplicate with three independent experiments (*n* = 9). Data represents means +/− SEM. * denotes significant difference between two groups, *p* < 0.05; ** denotes significantly higher than all other modalities, *p* < 0.05, # denotes control samples significantly lower than all other treatment modalities, *p* < 0.05)

RhBMP2_108 was then assessed for its ability to speed up osteoblast differentiation at various concentrations (Fig. 3, Fig. 4, Fig. 5, Fig. 6). First it was found that rhBMP2_108 was able to significantly increase ALP activity at seven and 14 days post seeding for all concentrations of rhBMP2_108 (Fig. 3). Interestingly, it was observed at 7 days that ALP activity was significantly higher at rhBMP2_108 concentrations of 50, 100 and 200 ng/ml when compared to control samples (Fig. 3). At 14 days, all treatment modalities receiving rhBMP2_108 at various concentrations demonstrated a marked 16-fold increase in ALP activity when compared to control samples void of rhBMP2_108 (Fig. 3).

Then MC3T3-E1 cells were assessed for mRNA levels by real-time PCR for genes encoding ALP, OCN, COL-1 and Runx2 at various concentrations of rhBMP2_108 (Fig. 4). For gene encoding Runx2, up to a five fold increase was observed at a concentration of 500 ng/ml when compared to control samples with a significant increase at 100 and 200 ng/ml compared to lower concentrations (Fig. 4a). Similarly, COL-1 also demonstrated concentration dependent increase in gene expression (Fig. 4b). Here however, only up to a two fold increase was observed relative to control samples (Fig. 4b). It was observed that mRNA levels of ALP were over five fold increased at 14 days at concentrations of ten and 50 ng/ml rhBMP2_108 whereas at a concentration of 100 and 200 ng/ml 20 fold increase in ALP activity (Fig. 4c). The highest concentration of 500 ng/ml demonstrated a 40 fold increase in ALP activity when compared to culture media without rhBMP2 (Fig. 4c). The greatest fold change was observed in OCN levels where up to a 300 fold increase was observed when compared to their respective control without rhBMP2 (Fig. 4d).

Lastly, alizarin red staining was used to visualize in vitro mineralization (Fig. 5). It was observed that without rhBMP2, MC3T3-E1 pre-osteoblasts were unable to generate any visual mineralization as observed by alizarin red staining (Fig. 5). The effects of rhBMP2_108 demonstrated a significant concentration dependent increase in alizarin red staining at 14 days post seeding (Fig. 6).

## Discussion

The aim of the present study was to investigate the bioactivity of a shorter recombinant BMP2 (rhBMP2_108) sequence on osteoblast behavior. The use of rhBMP2 for orthopaedic and dental treatments has been utilised for a number of procedures including open fractures, hip reconstructive surgeries as well as guided bone regeneration procedures in dentistry [30–33]. Despite demonstrating promising in vivo results, growth factor use has had a mix of success when utilised for clinical use. Some of the key concerns raised with respect to growth factor utilisation is their transient bioactivity which has been suggested could to be in the order of minutes to hours [18].

There are three main areas of research aimed at improving the bioactivity of growth factors; 1) utilising gene therapy, 2) improving protein stability or 3) improving the growth factor delivery system. One method that has shown early promise for growth factor delivery is that of gene therapy where the use of viral vectors such as adenovirus may circumvent many of the limitations of protein delivery by exhibiting a high in vivo transduction efficiency with a relatively short expression period [34–38]. Their main advantage is the over-expressed protein is constructed within the organisms main cell and thus has access to a multitude of folding proteins capable of accurately packaging and delivering growth factors to their surrounding tissues [25, 39, 40]. Despite the advancements made in this area of research, the use of gene therapy is still prohibited by the FDA making its future clinical use at the moment a future optimistic approach with no known timetable for its eventual use. Furthermore, recent strategies utilizing short synthetic BMP2-mimicking peptides have been shown to facilitate bone regeneration [41–44], however a lack of clinical reports utilizing such strategies is still lacking.

Thus it becomes vital to find alternative methods that may be deemed suitable for improvements in growth factor bioactivity. In the present study, a novel recombinant version of BMP2 (rhBMP2_108) with a shorter amino acid sequence. Prior to animal testing however, in vitro testing was performed on cell activity. It was found in the present study that r rhBMP2_108 was firstly able to target the recruitment of progenitor cells by favouring early and rapid recruitment of cells within 24 h (Fig. 1). The homing of progenitor cells and stem cells is one of the key initial stages in the early onset of fracture repair and is a necessary component of wound healing of bony-defects. As such, the ability for rhBMP2_108 to speed up the rate and number of progenitor cells to defects sites is crucial for bone repair.

Although the effects as seen in the present study demonstrated that rhBMP2_108 was significantly able to induce cell proliferation, it was not observed in a concentration dependent manner. This can be due to the fact that cells undergoing cell differentiation are not prone to proliferate simultaneously. While not much concentration dependant observations were found for cell recruitment or proliferation, significant differences were found for osteoblast differentiation in response to rhBMP2_108. It was observed that over a four fold increase in ALP activity were detected with rhBMP2_108 and further concentration dependant increases in alizarin red staining further validated our hypothesis that rhBMP2_108 acts primarily by stimulating osteoblast differentiation. It was also observed that a significant increase in mRNA levels of ALP was observed in a concentration dependant manner. Interestingly OCN, a late differentiation marker for osteoblasts was upregulated approximately 300 fold when compared to control samples (Fig. 4b). This finding likely signifies that the MC3T3-E1 pre-osteoblasts seeded in control wells without rhBMP2 likely remained progenitor cells and did not differentiate towards the osteoblast lineage throughout the course of these experiments. This is further evident by the fact that the samples without rhBMP2 showed no mineralization potential in the alizarin red experiments (Fig. 5).

Although the results from the present study confirm the effects of this shorter amino acid sequence of rhBMP2_108 on cell activity, much research remains necessary in order to validate these findings in an animal model prior to clinical testing. It remains virtually unknown if the protein activity will be affected in vivo by the shortened amino acid sequence and much future research investigating this relationship both with respect to protein half-life duration as well as its subsequent effects on bone formation need to be carefully evaluated. Furthermore, its comparison to leading rhBMP2 currently available on the market with FDA approval is also necessary to provide a better understanding on the effects of protein amino acid length on bioactivity of recombinant proteins.

## Conclusion

The results from the present study demonstrate that a shorter amino acid sequence of recombinant BMP2 (rhBMP2_108) retains bioactive properties of BMP2 when compared to commercially available rhBMP2 in vitro. It was shown that rhBMP2_108 was able to stimulate rapid cell recruitment of MC3T3-E1 pre-osteoblasts and support their differentiation towards the osteoblast lineage in a concentration dependant manner. Future in vivo research analyzing the use of this amino acid sequence of rhBMP2_108 is now necessary in critically-sized bone defects. Furthermore, investigation on the optimal delivery system this growth factor are necessary to potentially further improve its bioactivity for clinical use.

## Abbreviations

rhBMP2: recombinant bone morphogenetic protein 2
rhBMP2_108: Short polypetitde chain of 108 amino acids for recombinant bone morphogenetic proteins two
ALP: alkaline phosphatase
OCN: osteocalcin
COL-1: collagen 1
Runx2: runt-related transcription factor two
GAPDH: glyceraldehyde 3-phosphate dehydrogenase
FDA: food and drug administration
CHO: Chinese hamster ovary
PCR: polymerase chain reaction
PBS: phosphate buffered solution
HCl: hydrochloric acid
EDTA: ethylenediaminetetraacetic acid
NaCl: sodium chloride
DMEM: Dulbecco’s modified eagle medium
BCA: bicinchoninic acid
OD: pptical density
SE: standard error
